# Challenges of Pediatric Cataract Surgery in a Case of Seasonal Hyperacute Panuveitis (SHAPU)

**DOI:** 10.1155/2021/5591859

**Published:** 2021-08-27

**Authors:** Pratap Karki, Ranju Kharel Sitaula, Anadi Khatri, Sagun Narayan Joshi, Haramaya Gurung, Indraman Maharjan, Ananda Kumar Sharma, Madan Prasad Upadhaya

**Affiliations:** ^1^Department of Ophthalmology, Maharajgunj Medical Campus Tribhuvan University, Institute of Medicine, B. P. Koirala Lions Centre for Ophthalmic Studies, Maharajgunj, Kathmandu, Nepal; ^2^Department of Ophthalmology, Maharajgunj Medical Campus, Institute of Medicine, Tribhuvan University, B. P. Koirala Lions Centre for Ophthalmic Studies, Maharajgunj, Kathmandu, Nepal; ^3^Birat Medical College and Teaching Hospital Biratnagar, Nepal; ^4^Dept of Ophthalmology, Maharajgunj Medical Campus, Tribhuvan University, Institute of Medicine, B.P. Koirala Lions Centre for Ophthalmic Studies, Maharajgunj, Kathmandu, Nepal; ^5^Himalaya Eye Hospital, Pokhara, Nepal; ^6^Department of Ophthalmology, Maharajgunj Medical Campus, Institute of Medicine, B.P. Koirala Lions Center for Ophthalmic Studies, Nepal; ^7^Ophthalmology, Tribhuvan University, Chair Emeritus B.P Eye Foundation, Children Hospital for Eye, ENT and Rehabilitation (CHEERS), Bhaktapur, Nepal

## Abstract

A four-year-old female child diagnosed as a case of severe Seasonal Hyperacute Panuveitis (SHAPU) underwent lens-sparing core vitrectomy in her left eye with intravitreal antibiotic and steroid. Patient responded well to treatment and intraocular inflammation subsided. However, three months later, she developed vision impairing dense cataract which also made posterior segment assessment difficult. Lens aspiration with primary posterior capsulotomy and anterior vitrectomy with intraocular lens (IOL) implantation was performed. However, four weeks later, the patient developed occlusio pupillae with iris bombe. She did not respond to medical management so synechiolysis with surgical iridectomy was performed after which a normal depth anterior chamber was attained. Synechia and iris bombe were also relieved, and vision was regained.

## 1. Introduction

Seasonal Hyperacute Panuveitis (SHAPU) is rare but devastating panuveitis reported from Nepal since last 46 years in autumn of every odd year. Most cases have been reported from hilly regions in and around Kaski district of Nepal. Association has been seen with direct physical exposure or vicinity to the white moths [1]. History of physical contact with moth was seen in 48.6% of SHAPU cases and 11.4% of control in a case control study [1]. It causes uniocular blindness and is the commonest cause of panuveitis among the Nepalese children [1–3]. In a previous study, 71.4% cases were children [1]. The role of white moths in SHAPU causation is still unclear, and studies are being conducted to evaluate, whether the SHAPU is due to the auto-immune response to the moth toxins or due to pathogens delivered by the moths over the ocular surface. Similarly, why it primarily affects children is still not understood.

Sudden onset of red eye with severe intraocular reactions, leukocoria, and hypotony are the characteristic features of SHAPU. No standard treatment protocol for SHAPU has been developed over the past 46 years. So, for the wider coverage of both microbial and immunological aspects, a blanket therapy with antibiotics and steroid is used. The periocular injections and ocular surgery are carried depending upon the grading of SHAPU [4]. As an ocular emergency, severe SHAPU needs urgent intravitreal injections and vitrectomy with or without lensectomy. Visual rehabilitation after lensectomy in any SHAPU child remains a challenge. Herein, we report a SHAPU case which received immediate management with core vitrectomy but developed cataract for which she underwent intraocular lens (IOL) implantation. Soon she developed unexpected complications in the form of occlusio pupillae and iris bombe and presented as secondary angle closure with ocular hypertension. This is the first report of its kind related to SHAPU. The written consent from the legal parents has been obtained for the publication.

## 2. Case Report

**Figure 1:**
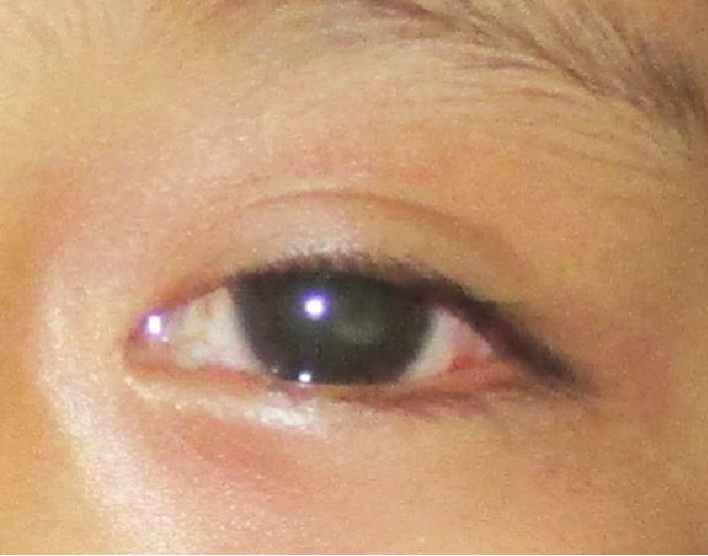
Presence of congestion and leukocoria in the left eye.

**Figure 2:**
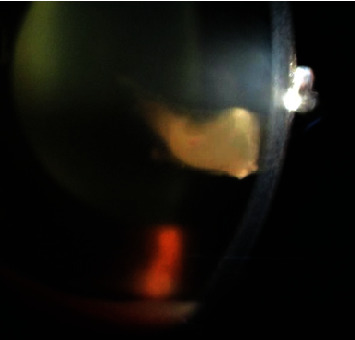
Slit view of the left eye showing an exudative clump in the middle of anterior chamber along with 1 mm cream colored settled hypopyon.

**Figure 3:**
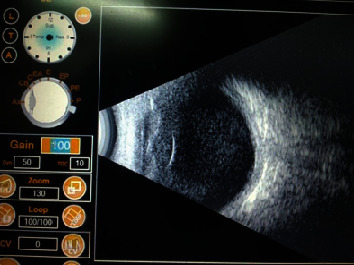
USG (B) scan showing multiple hyperechoic opacities in the mid and postvitreous with an attached retina.

A 4-year-female child from Kaski district, Pokhara, presented with sudden onset of redness, diminution of vision, and watering in her left eye for 2 days (Figure 1) on 10th September 2019. Pokhara is the same region which is known to have many reported cases of SHAPU in every SHAPU epidemic. On specific inquiry, there was history of sudden increase in white moth population in their area. However, there was no history of direct exposure of the child to the moths. The child had no complaints of pain and was cooperative during assessment. Visual acuity (VA) in her right eye was 6/6, N6. In the left eye, VA was hand movement close to the face with inaccurate projections of rays. Examination of the affected eye revealed circumcorneal congestion and clear cornea with dense anterior chamber reaction. There was a clump of thick exudates floating in the middle of anterior chamber along with 1 mm cream colored settled hypopyon (Figure 2). Leukocoria was present, and the fundal glow and fundus details were not visible. Ultrasound brightness (B) scan revealed dense vitreous hyperechoic opacities in the mid and postvitreous with thickened choroid. Echogenic impression revealed an attached retina (Figure 3). Intraocular pressure (IOP) was 8 mmHg with air puff tonometry. Her right eye was found to be normal in all aspects; routine blood investigations were normal.

**Figure 4:**
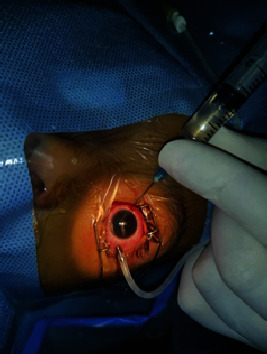
Diagnostic vitreous tap of the left eye showing light yellow aspirate in the syringe.

With the diagnosis of left eye severe SHAPU, the child immediately underwent lens-sparing core vitrectomy under general anesthesia. Three sclerotomies were made 4 mm from the limbus, and the 20 gauge vitrectomy was performed at 1500 cuts/minute with vacuum at 350 mmHg. After removing core vitreous, the posterior hyaloid was left intact. Intraoperatively, no retinal necrosis was seen. Fluid air exchange was done at the end of procedure, and the sclerotomy sites were closed under air tamponade at 30 mmHg. Prior to the vitrectomy, diagnostic aqueous tap with 26 G needle and vitreous tap with 3 ml syringe was done (Figure 4). And at the end of vitrectomy, intravitreal antibiotics (vancomycin 1 mg/0.1 ml + ceftazidime 2.25 mg/0.1 ml) and intravitreal steroid dexamethasone (0.4 mg/0.1 ml) were given. The retina appeared intact so only air tamponade was used. After vitrectomy, the child was under moxifloxacin eyedrop and prednisolone acetate eye drop hourly and atropine thrice daily. Systemic antibiotic injection (ceftriaxone 250 mg × BD × 7 days) and oral steroid (1 mg/kg body weight) were administered on tapering dose. Diagnostic aqueous and vitreous tap did not reveal any microbial organisms on Gram, Giemsa, and KOH stain. The ocular fluid samples and blood samples, none grew any organism in Blood agar, Brain Heart Infusion broth, and Sabouraud dextrose agar.

By seventh postoperative day, her vision improved to 5/60 N10, the circumcorneal and conjunctival congestion reduced, hypopyon and exudates disappeared, and cellular reactions in anterior chamber decreased. Fundal glow was visible, but details could not be appreciated due to vitritis and onset of cataract.

On subsequent regular follow-up, her intraocular inflammation resolved, and her eye became quiet after 3 months, but her cataract gradually became dense in the left eye; fundus was not visible, but vitreous was clear with attached retina on USG B Scan. Her vision deteriorated to 2/60 N10; however, posterior segment appeared normal in repeat B scan.

After next 8 weeks quiescence of intraocular inflammation, she underwent lens aspiration with 3 mm primary posterior capsulotomy followed by anterior vitrectomy and primary intraocular lens implantation (PMMA-Fred Hollows Foundation Lens) in the bag without optic capture under general anesthesia. She was started on oral steroid at 0.5 mg/kg/body weight 7 days prior to surgery. Postoperatively, she had VA of 6/36 N10, and oral steroid was tapered over 3 weeks.

**Figure 5:**
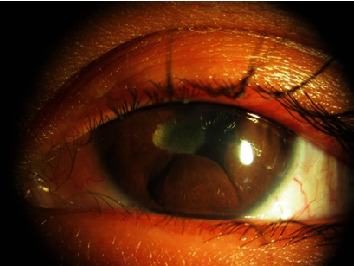
Occlusio pupillae with iris bombes at multiple areas in the left eye.

**Figure 6:**
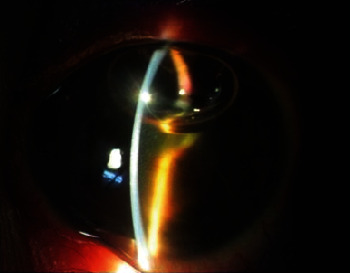
Slit lamp view of the left eye showing relieve of iris bombe after synechiolysis but presence of postoperative inflammatory reactions in the anterior chamber.

But one month after the cataract surgery, she presented with painful red eye, and examination revealed occlusio pupillae with iris bombe and shallow anterior chamber in the operated eye (Figure 5). The IOP was 22 mmHg with Goldmann applanation tonometer. She was kept under hourly prednisolone acetate 1% eyedrop, atropine thrice daily, and beta blockers twice a day. Subconjunctival injection of mydricaine was given in four quadrants. But no sign of relief of iris bombe noted. Next day, again under repeat general anesthesia, she underwent 360° synechiolysis with peripheral iridectomy (PI) at 2 o'clock with vitrectomy cutter (Figure 6). Intraocular lens was in situ at bag and was not removed during surgery. On 1st postoperative day, the child was comfortable with quiet eye, vision was 5/60 N10, anterior chamber was normal, PI was patent, intraocular lens was in position, and the fundus was normal. Her IOP was 12 and 13 mmHg without any antiglaucoma drugs. Her best corrected visual acuity in 6 months follow-up was 6/60 N10 and is under amblyopia therapy for visual rehabilitation.

## 3. Discussion

SHAPU causes severe intraocular inflammation which if not treated and controlled in time leads to permanent visual loss and hypotony. Mild to moderate cases respond well to timely intravitreal injection of antibiotics and steroid [4]. However, severe cases require core vitrectomy in combination with intravitreal injections.

Use of steroids and vitrectomy may predispose these cases to develop complicated cataract. Inadvertent lens touch can also cause cataract, although no so such event was noted in our case. Early cataract may sometimes be managed conservatively, and surgery can be delayed. Dense cataract however warrants early surgery not only for routine evaluation of the posterior segment but also to prevent amblyopia as SHAPU happens mostly in young children.

Decision for complete vitrectomy with primary lensectomy in a child with SHAPU has to be done judiciously. Entire removal of the vitreous with lensectomy may be fruitful to prevent subsequent recurrence and proliferative vitreoretinopathy. On the other hand, core vitrectomy without removal of cortical vitreous can be enough to remove the bulk of vitreous exudates without untoward damage to the fragile retina. Likewise, lensectomy at the same sitting can help to increase visibility during vitrectomy, but lens-sparing vitrectomy also proves worthy to prevent postoperative amblyopia in these young children [4].

Challenges of cataract surgery in patients with uveitis are well known. Good preoperative inflammation control is a prerequisite for surgery in these patients, and postoperative inflammation also needs to be controlled well [5]. However, in our case despite good preoperative inflammation control, patient developed postoperative complication resulting in synechia and iris bombe. This is a known postoperative complication of cataract surgery in patient with uveitis but had not been reported in any SHAPU cases earlier [6].

Severe inflammation associated with SHAPU especially in young children often predispose to severe postoperative inflammation and complications [7]. This poses a challenge when doing cataract surgery in these children with additional the threat of complications related to general anesthesia.

Primary vs. secondary intraocular lens implantation in pediatric uveitis cataract has been the topic of debate over decades. Doubtful outcome has been reported in uveitis cases with primary intraocular lens implantation [7]. We implanted the primary intraocular lens in this case for early visual rehabilitation and for prevention of amblyopia. But, our experience with this case has shown better to avoid primary intraocular lens to reduce further intraocular inflammation-related complications in a SHAPU case.

## 4. Conclusion

Cataract surgery can have severe postoperative inflammation in children with SHAPU despite good preoperative control. Our experience in this case suggests avoiding primary intraocular lens implantation while doing cataract surgery in these patients.
